# The chaperone-like activity of the hepatitis C virus IRES and CRE elements regulates genome dimerization

**DOI:** 10.1038/srep43415

**Published:** 2017-02-24

**Authors:** Cristina Romero-López, Alicia Barroso-delJesus, Alfredo Berzal-Herranz

**Affiliations:** 1 Instituto de Parasitología y Biomedicina López-Neyra, IPBLN-CSIC, PTS Granada, Avda. del Conocimiento 17, 18016 Armilla, Granada, Spain; 2 Unidad de Genómica, Instituto de Parasitología y Biomedicina López-Neyra, IPBLN-CSIC, PTS Granada, Avda. del Conocimiento 17, 18016 Armilla, Granada, Spain

## Abstract

The RNA genome of the hepatitis C virus (HCV) establishes a network of long-distance RNA-RNA interactions that direct the progression of the infective cycle. This work shows that the dimerization of the viral genome, which is initiated at the dimer linkage sequence (DLS) within the 3′UTR, is promoted by the CRE region, while the IRES is a negative regulatory partner. Using differential 2′-acylation probing (SHAPE-dif) and molecular interference (HMX) technologies, the CRE activity was found to mainly lie in the critical 5BSL3.2 domain, while the IRES-mediated effect is dependent upon conserved residues within the essential structural elements JIIIabc, JIIIef and PK2. These findings support the idea that, along with the DLS motif, the IRES and CRE are needed to control HCV genome dimerization. They also provide evidences of a novel function for these elements as chaperone-like partners that fine-tune the architecture of distant RNA domains within the HCV genome.

RNA viral genomes show hierarchical structural organization. The primary structure encodes proteins, while the secondary and tertiary structures code for essential viral regulatory mechanisms^1^. Indeed, highly conserved structural RNA elements that recruit cellular and viral proteins, and that establish dynamic, functional, long-distance RNA-RNA contacts^1^, control the transition between translation, replication and encapsidation: the genome itself thus provides the molecular regulators that direct some of the most critical events of the infective cycle.

Hepatitis C virus (HCV) genome is a positive ssRNA molecule that encapsidates as a single copy in the mature virion. It presents highly conserved structural elements^2^,^3^,^4^,^5^,^6^. At the 5′ end, the internal ribosome entry site (IRES) directs the initiation of translation. It is composed by a set of stable stem loops connected by complex three- and four-way junctions (Fig. 1a)^7^,^8^. IRES activity is dependent upon domains II, III and IV^9^,^10^, which bear all the structural requisites for directly positioning the 40S ribosomal subunit at subdomain IIId^11^,^12^,^13^,^14^ and directing the conformational rearrangements necessary to fit the translation start codon into the ribosomal P site^14^,^15^,^16^. Though the eIF3 translation initiation factor is also recruited by the IRES at the IIIabc four-way junction (JIIIabc)^17^,^18^,^19^, it is not essential for the initiation of translation *per se*^20^. The translation process is additionally regulated by distant functional genomic RNA domains, such as the 3′UTR^21^,^22^ and the *cis*-acting replication element (CRE)^23^.

The 3′UTR has a key role in replication^24^, encapsidation^25^ and infectivity^26^. It also enhances long-term IRES function by aiding in the recruitment of the 40S ribosomal particle to the highly conserved 3′X-tail^27^. This tail adopts one of two conformations^28^,^29^ (Fig. 1a): i) a three stem-loop isoform – 3′SLI, 3′SLII and 3′SLIII^30^, or ii) a two stem-loop conformer in which 3′SLI is preserved while the 3′SLII and 3′SLIII domains fold back into a single stem-loop, exposing a palindromic, highly conserved sequence motif, the so-called dimer linkage sequence (DLS; Fig. 1a)^29^,^31^. HCV genomic dimerization can be initiated at the DLS by the formation of a kissing complex between the nucleotides exposed in the loops^32^. In the absence of protein cofactors, such unstable contact can evolve, under certain conditions, towards an extended duplex conformation involving the whole DLS motif^33^. Recent reports have shown that this dimeric complex is an optimal template for viral RNA-dependent RNA polymerase^34^, but its contribution to other steps in the viral cycle remains unclear.

The CRE region is a critical regulatory partner in virus propagation. It functions as a negative regulatory agent during viral translation^23^ and as a replication enhancer^35^,^36^. It consists of three conserved domains, 5BSL3.1, 5BSL3.2 and 5BSL3.3 (Fig. 1a). The 5BSL3.2 is the core nucleation centre of a network of RNA-RNA contacts (Fig. 1a): i) its apical loop interacts with the apical loop of 3′SLII at the 3′X-tail^37^,^38^; this interaction overlaps the DLS motif and may be incompatible with the acquisition of the dimerizable conformation^29^; ii) the 5BSL3.2 bulge can either interact with a conserved sequence centred on nucleotide 9110 (the so-called Alt motif)^39^,^40^ or make an equally stable contact with the apical loop of the subdomain IIId in the IRES^41^. Interestingly, the contact IIId-5BSL3.2 would render circular forms of the HCV genome mediated by direct RNA-RNA contacts, similar to that which occurs in flaviviruses and picornaviruses^1^,^40^,^41^,^42^,^43^. The establishment of such topologies is essential for controlling multiple steps of the infective cycle and provides important benefits, such as an increase in the local concentration of essential proteins and cofactors, and additional protection against the action of exonucleases. The contacts 3′SLII-5BSL3.2 and Alt-5BSL3.2 are required for efficient viral RNA synthesis^37^,^39^ and translation^44^, while the long-range interaction IIId-5BSL3.2 operates as a negative regulatory agent of HCV translation^23^. This network of interactions also promotes conformational rearrangements, not only in the domains directly involved, but also in surrounding areas. It therefore acts as an RNA structural organizer^42^,^43^. This property has important consequences for the functionality of the affected viral genomic regions. We previously described that the acquisition of the dimerizable conformer in the HCV RNA genome is dependent, under suboptimal conditions, on the presence of the IRES plus the CRE^43^, which points to a yet unknown contribution of the IRES and CRE regions to HCV dimer formation.

The present manuscript explores the molecular requirements for HCV dimerization apart from those described for the 3′X-tail, with the aim of establishing links between the interactions IRES-CRE-3′X and their potential involvement in the viral cycle. Electrophoretic mobility shift assays showed that dimer formation efficiency is regulated by both the IRES and CRE regions in an opposing manner. High-throughput structural analyses based on SHAPE technology showed specific residues located in essential regions of the IRES and the CRE (such as subdomain IIId or the 5BSL3.2 element) to be strongly involved in dimer formation and in fine-tuning the folding of the dimer. Taken together, these results show that viral dimerization is not only dependent on the formation of a kissing complex at the 3′X-tail, but also that it is governed and influenced by distant, conserved elements of the viral genomic RNA. To our knowledge, this is the first report providing evidence of the potential of the IRES and CRE regions providing an organizing centre in HCV genomic dimerization.

## Results

### The CRE and the IRES regions influence genomic dimer formation

HCV genome dimeric complex formation is initiated at the highly conserved palindromic sequence motif DLS in the 3′X-tail^29^,^31^. This process occurs in the absence of any coadjuvant^32^, but it can be implemented and stabilized by the presence of the viral core chaperone protein^29^,^31^,^32^. It has also been reported that both the IRES and CRE may determine the acquisition of a dimerizable conformer under suboptimal conditions^42^,^43^. However, the impact of such structural rearrangements on the HCV dimerization event itself remains unknown.

**Figure 1:**
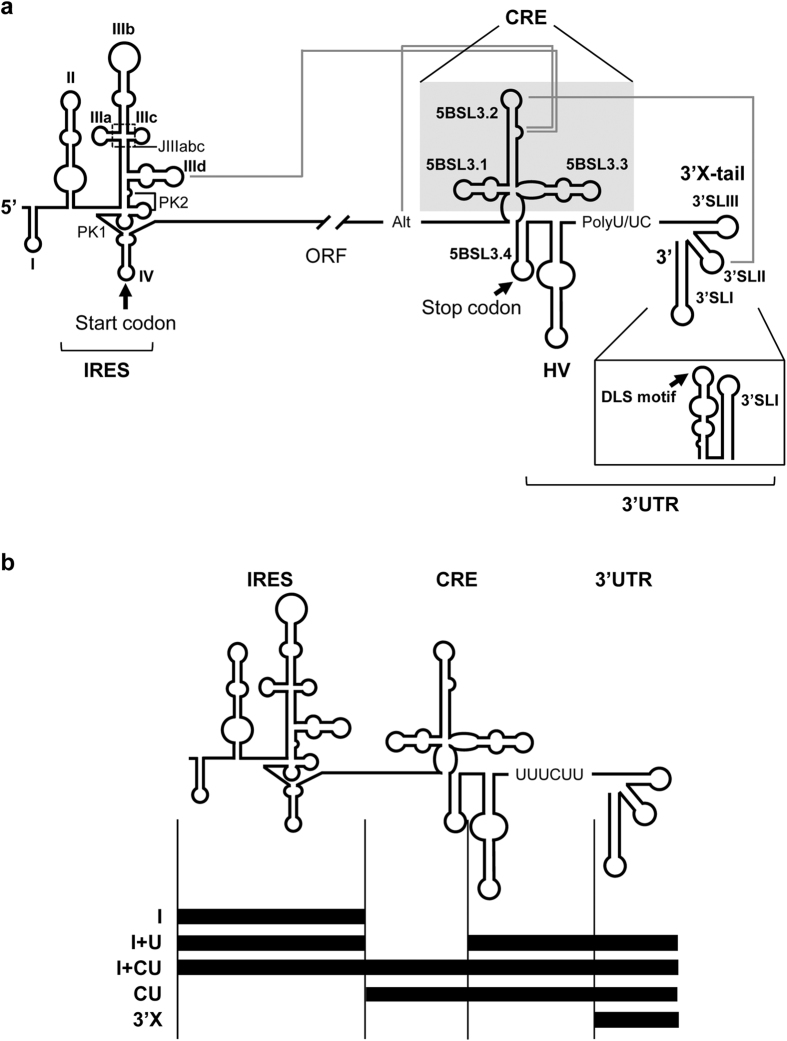
Secondary structure of the 5′ and 3′ ends of the HCV genome. (**a**) Diagram of the secondary structure at the 5′ and 3′ termini of the HCV genome. Functional RNA domains are indicated. The CRE region is shadowed. The 3′X-tail folds into two different conformers. Pseudoknot elements within the IRES are marked as PK1 and PK2. JIIIabc denotes the four-way junction organizing the stem loops IIIa, IIIb and IIIc in the IRES. The dimerization linkage sequence (DLS) and start and stop translation codons are indicated by arrows. The grey solid lines identify long-distance RNA-RNA interactions involving the IRES, the Alt sequence, the CRE and the 3′X-tail regions. (**b**) Diagram of the series of HCV transcripts employed in this work.

**Figure 2:**
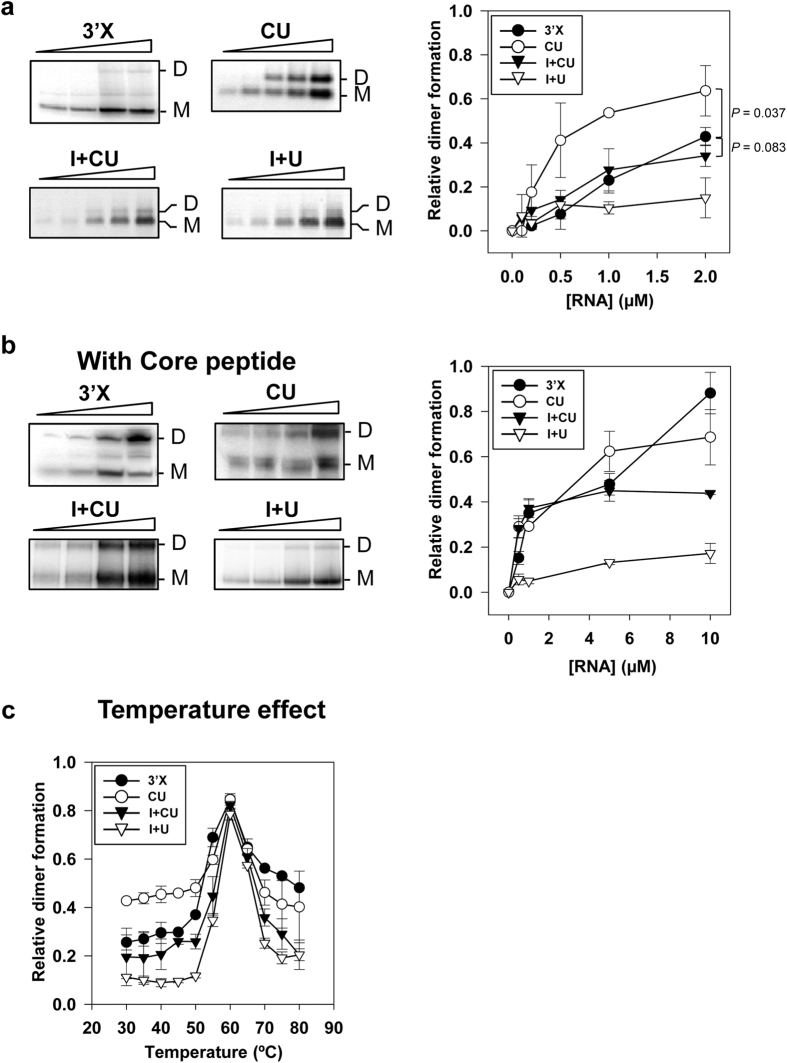
Differential dimerization ability of the 3′X-tail induced by the IRES and the CRE. (**a**) Left panel, dimerization process efficiency was monitored at different concentrations of 3′X, CU, I+CU and I+U. Dimeric products were resolved by native agarose (for CU, I+CU and I+U) or polyacrylamide gel electrophoresis (for 3′X). A representative image of the electrophoretic mobility shift assays is shown. Relative dimer formation for each transcript was quantified (right panel). Data represent the mean of at least three independent experiments ± standard deviation. (**b**) Core protein addition strongly promoted dimer formation for transcript 3′X, and to a lesser extent for CU, I+U and I+CU. Dimerization assays were performed in the presence of the core 2BD peptide at a protein to nucleotide molar ratio of 1:50. After 15 min incubation at 37 °C, the core protein was removed by digestion with proteinase K. RNA complexes were resolved by native polyacrylamide gels. A representative dimerization experiment for each transcript is shown in the left panel. Relative dimer formation was quantified (right panel). Data are the mean of three independent assays ± standard deviation. (**c**) Dimeric complexing reached maximum efficiency at high temperatures for all the transcripts tested. Fixed amounts of the transcripts were tested for their ability to dimerize at different temperatures. Reaction products were processed and analyzed as mentioned above. Values are the means of three independent assays ± standard deviation.

Dimerization assays were performed with the previously reported transcripts CU^41^ and I+CU^43^ (Fig. 1b). These molecules respectively encompass the CRE region fused to the 3′UTR, and the IRES element plus the CU construct. Control reactions were performed with the 3′X construct containing the 3′X-tail region, which can dimerize to some extent^32^ (Fig. 1b). Increasing concentrations of the RNA constructs were incubated for 15 min at 37 °C under low magnesium conditions to achieve dynamic folding^43^,^45^. Dimeric and monomeric isoforms were resolved as described in Materials and Methods. A first overview revealed CU to show a significant increase (*P* ≤ 0.05) in dimerization efficiency compared to the 3′X transcript (Fig. 2a). Thus, the direct interaction between the CRE and the 3′X-tail promotes dimer formation, most likely by favouring optimal conformation for the exposure of the DLS motif in an apical loop. Interestingly, the addition of the HCV IRES element (I+CU) significantly (*P* ≤ 0.05) repressed the enhancement of dimer formation promoted by the CRE region (Fig. 2a), yielding dimerization levels close to those obtained for the 3′X molecule. This effect might be due to an indirect action resulting from the interaction of the IRES with the CRE region^41^, which might sequester the dimerization enhancer motifs present in the CRE, or it might be the result of a direct effect, the consequence of a yet unknown interaction between the IRES region and the 3′X-tail. To examine the direct role of the IRES element in HCV dimerization, the transcript I+U, which lacks the CRE region, was subjected to dimer formation assays as noted above (Fig. 2a). This revealed the IRES to act as a potent negative regulator of the dimerization event, with inhibition values of around 65% attained compared to those returned by the 3′X-tail alone (Fig. 2a).

Taken together, these data show that the HCV genome dimerization is governed by conserved functional domains distinct from the DLS motif, located in the IRES and CRE regions.

### Long-distance RNA-RNA contacts are involved in HCV genome dimerization

It seemed feasible that the effect promoted by the IRES in genome dimerization might be the result of the acquisition of non-dimerizable RNA conformations. These unproductive structures might collapse further transitions towards dimerization-efficient architectures, which could then be released by the addition of chaperone proteins. It has been described that the HCV core protein promotes and directs the dimerization of the 3′UTR *in vitro*^31^ by inducing significant structural rearrangements in the 3′X-tail that achieve the exposure of the DLS in an apical loop^29^,^31^,^32^. This core protein is a 191 amino acid-long polypeptide^46^ with a hydrophilic, highly basic N terminus with RNA and protein binding properties located in well-defined basic clusters^29^,^47^. RNA chaperone functionality is retained in the second and third basic clusters of the N-terminal end (the so-called 2BD peptide)^29^. Therefore, the synthetic peptide 2BD was used to evaluate the role of this viral chaperone on the dimerization efficiency of the transcripts under study. Increasing amounts of the CU, I+CU, I+U or 3′X RNAs were incubated with SHAPE buffer and 2BD for 15 min. After protein removal by treatment with proteinase K, samples were resolved in native polyacrylamide gels (Fig. 2b). Dimer formation efficiency by the 3′X construct was significantly improved (*P* ≤ 0.05) in the presence of the viral chaperone, confirming previously reported data^29^,^31^,^32^. For CU, I+CU and I+U, the addition of the 2BD core peptide did not substantially improve the dimer ratio with respect to the results obtained in the absence of the chaperone protein (Fig. 2b). This result suggests that the IRES and/or the CRE promote structural rearrangements in the 3′X-tail that cannot be overcome by the core chaperone peptide. These structures may operate as ‘conformational traps’ for HCV genomic dimerization.

The secondary and tertiary structures of viral genomes are highly dependent on temperature. In retroviruses such as HIV, it is reported that the initial kissing complex formed during genomic dimer formation can evolve towards a stable duplex at 55 °C^48^. This progression should occur by destabilization of the basal portion of the stem, which lowers the energy barrier for the formation of the extended duplex without disruption of the loop-loop interaction^49^. The effect of the temperature on dimer formation was monitored for the RNA transcripts under study in order to examine the contribution of the IRES and CRE on the stability of the duplex. For this, a concentration of 1 μM of each transcript was incubated in SHAPE buffer for 15 min at temperatures ranging from 30–80 °C. The results showed all the RNA molecules to have a similar dimer formation yield at 55–60 °C, the optimal temperature range for duplex constitution (Fig. 2c). However, at high temperatures (65–80 °C), the IRES and the CRE reduced the dimer stability obtained with the 3′X transcript to different extents. While the CRE region barely affected the dimer ratio (transcript CU; Fig. 2c), the IRES alone promoted a strong reduction in dimer stability (molecule I+U), again supporting the existence of a direct IRES-3′X contact. Interestingly, the instability promoted by both regions was not additive; rather, it was partially balanced when the CRE and the IRES were both present (transcript I+CU).

These results suggest the existence of a long-range contact network involved in the control of dimerization. These interactions involve the IRES, the CRE and the 3′X-tail. The results also show that distant genomic RNA elements affect, either directly or indirectly, the conformation of the dimer, and more importantly, its thermodynamic properties.

### The conformation of the 3′X-tail is defined by the IRES and CRE

Regulation of HCV dimerization and dimer stability by the IRES and CRE regions is likely mediated by a conformational rearrangement, either promoted by a yet unknown direct IRES:3′X-tail interaction, or by an indirect effect derived from the contact IRES:CRE influencing the folding of the functional RNA domains required for the acquisition of the dimerizable isoform^42^,^43^. In fact, both systems may be involved.

Structural analysis of the dimeric conformer was performed using the SHAPE-dif method^50^ for the transcripts I+CU, CU and I+U. This makes use of the singular reactivity features of certain chemical reagents, such as the conventionally used SHAPE reagent NMIA^51^ or 1M6 (1-methyl-6-nitroisatoic anhydride). While NMIA preferentially reacts with residues that stabilize the architecture of the RNA molecule at small timescales (slow electronic dynamics)^52^, the second probe attacks “one-sided” stacked nucleotides, a typical conformation seen in bulges, turns, closing helix pairs, and long-range stacking interactions (fast electronic dynamics)^50^. Compilation of the reactivity data obtained with both reagents renders the fingerprinting map, which summarizes the non-canonical and stacking interactions defining the three-dimensional architecture of the RNA molecule.

**Figure 3:**
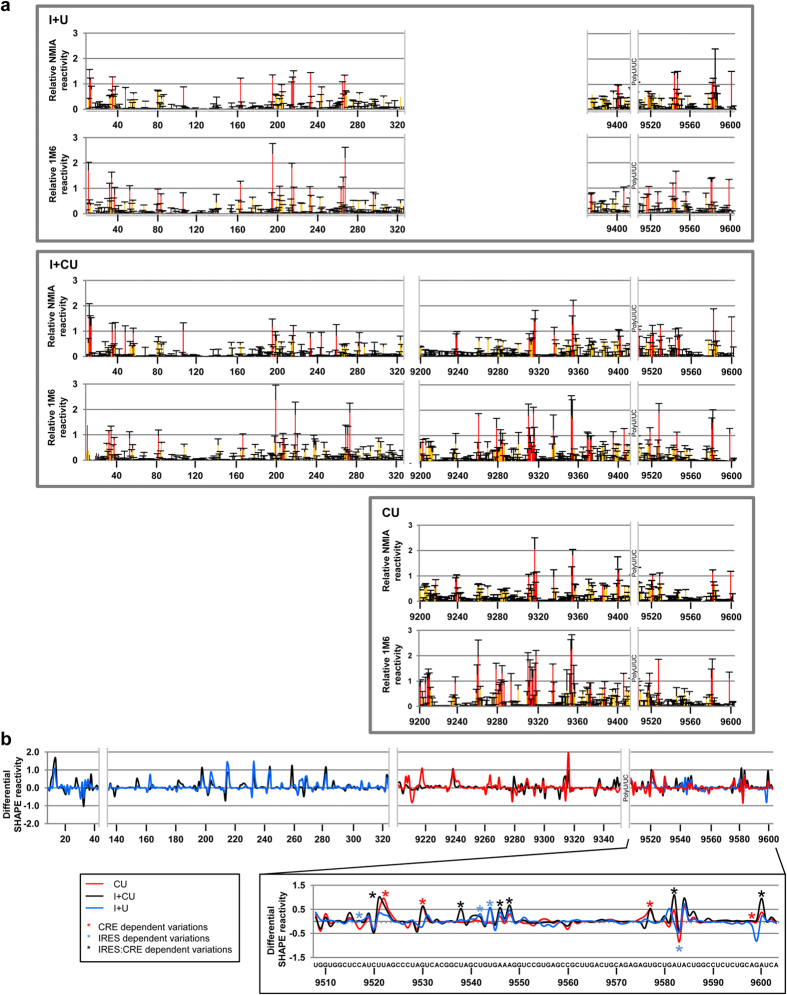
SHAPE-dif shows structural differences in the dimeric complex induced by the IRES, the CRE, and the 3′UTR. (**a**) The folding of CU, I+CU and I+U was analyzed by differential SHAPE reactivity (SHAPE-dif). Histograms show normalized SHAPE reactivities from reactions with NMIA (top) and 1M6 (bottom) for each transcript, coloured by relative nucleotide reactivity. Values are the mean of at least three independent experiments ± standard deviation. (**b**) The line graph shows the differential SHAPE reactivities calculated by scaling 1M6 to NMIA relative values over a sliding window and then subtracting corrected 1M6 from NMIA reactivity data. Strong differential observations (>| 0.5 | reactivity units) specific for each HCV genomic region – IRES, CRE or 3′UTR - are denoted by black, red and blue asterisks respectively.

A preliminary overview of the differential reactivity pattern for the dimerization competent molecule CU showed that a significant number of residues bear reagent specificity (Fig. 3). Interestingly, the nucleotides included in the apical loops of the 3′SLI, 3′SLII and 3′SLIII domains showed strong selectivity for NMIA (3′SLIII and 3′SLII, positions 9520–9530 and 9570–9580 respectively) or 1M6 (positions centred in 9580). In addition, the marked reduction (~0.75 reactivity units) seen in the SHAPE-dif pattern around positions 9280–9300, corresponding to the 5BSL3.2 apical loop (Fig. 3b), is consistent with the existence of stacking interactions related to the establishment of tertiary RNA-RNA contacts, most likely with the distant 3′SLII element in the 3′X-tail^37^,^38^. Strong oscillations in the SHAPE-dif profile were also noted in the regions surrounding the apical loops of domains 5BSL3.1 and 5BSL3.3 (positions 9240 and 9320 respectively), again suggesting preferential structural dynamics in these elements.

**Figure 4:**
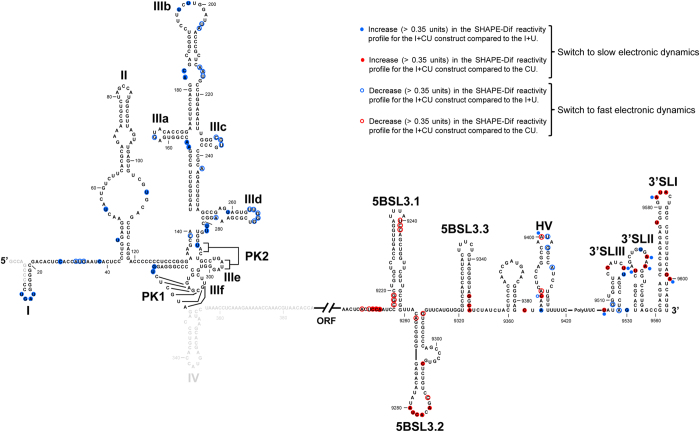
The IRES and the CRE tune the HCV RNA conformation under dimeric conditions. Comparison of the results obtained by SHAPE-dif for I+CU with respect to CU and I+U. Filled circles: significant increase (>0.35 units, *P* ≤ 0.05) in the SHAPE-dif reactivity profile for I+CU compared to CU (red) and I+U (blue); open circles: significant reduction (>0.35 units, *P* ≤ 0.05) in the SHAPE-dif reactivity profile for I+CU compared to CU (red) and I+U (blue).

The I+CU transcript, which has limited dimerization capacity, showed a different reactivity profile for certain nucleotides with respect to CU, supporting the role of the IRES in tuning the structure of the 3′ end of the HCV RNA (Fig. 3b)^43^. In the 3′X-tail, this was strongly evidenced at the critical U9538, an essential nucleotide for the initiation of the kissing complex during dimer formation^32^. It was also noted that the SHAPE-dif profile of the I+CU molecule substantially changed around the apical loop and the bulge of the 5BSL3.2 domain (Figs 3b and 4). This disturbance can be linked to the long-range interaction 5BSL3.2:subdomain IIId^41^, which might be related to the acquisition of a dimerizable isoform. Finally, the SHAPE-dif profiles for I+CU differed to that shown by CU at the base of the 5BSL3.1 domain (positions 9210–9220 and 9262; Fig. 3) flanking the stem-loop. This region may act as a hinge that allows the acquisition of a cruciform architecture in the CRE region, which might be required for efficient replication^35^,^36^. As well as the specific changes in the relative reactivity pattern, common trends were noticed in the SHAPE-dif pattern for the CU and the I+CU constructs at positions centred around nucleotides 9520, 9530, 9576 and 9600 (*P* < 0.05; Fig. 3b, lower panel, red asterisks). This behaviour confirms that the CRE region contributes towards the preservation of the three-dimensional folding at the 3′ end of the viral genome^37^,^38^.

SHAPE-dif analysis was performed with I+U to discern whether the IRES itself plays a role in the 3′X folding. The resulting data provided an interesting conclusion: the IRES region influences the conformation of the 3′X-tail in the absence of the CRE (Figs 3b and 4). This can be clearly seen from the SHAPE-dif profiles at positions C9517, U9544, G9545 or U9583. In these residues, the presence of the IRES constrains the SHAPE-dif pattern (compare the I+U and I+CU profiles to that of CU; Fig. 3b), supporting the idea of the IRES having a direct effect on the conformation of the 3′X-tail. This result also provides structural clues for understanding the inhibitory effect of the IRES region in dimerization.

Taken together, the results provide solid evidence of the role of the CRE and IRES regions in the conformational rearrangement of the 3′X-tail. Such structural modifications may be critical for regulating the acquisition of the dimerizable isoform.

### Identification of residues required for HCV dimerization

A modification interference strategy^53^ was used to examine which residues of the conserved functional long distant RNA domains are required for the acquisition of the dimer isoform with the I+CU (low dimerization efficiency) transcript. HMX methodology (2′-hydroxyl molecular interference) was followed, employing 2′-hydroxyl selective SHAPE chemistry^54^. NMIA was chosen as a modifying agent since it works well at high temperatures by introducing bulky adducts that interfere with RNA folding in particularly crowded regions, such as those where RNA-RNA tertiary contacts occur^54^. I+CU transcript modified under denaturing conditions was folded to promote dimerization events and subjected to resolution by native polyacrylamide gel electrophoresis. Monomeric and dimeric conformations were identified and NMIA modifications were detected by primer extension. The resulting reactivity data were cross-correlated to calculate the HMX score^54^, thus identifying those residues preferentially modified in the monomer or dimer (Methods).

**Figure 5:**
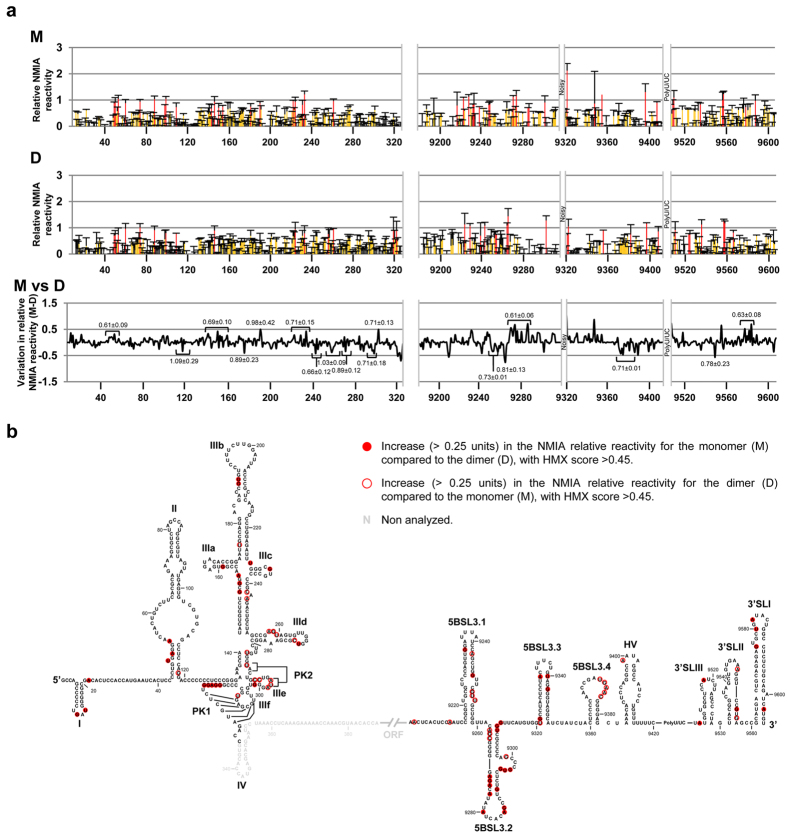
Identification of nucleotides influencing dimer formation in the HCV genome. Nucleotides required for dimer formation were identified by 2′-hydroxyl molecular interference (HMX). (**a**) Molecule I+CU was modified with NMIA under denaturing conditions. The monomeric and dimeric populations were then partitioned by native polyacrylamide gel electrophoresis. Modified positions were detected as stops in a reverse transcription reaction. Histograms show the NMIA reactivity profile for each of the isolated pools, M (monomer) and D (dimer). Data are the mean of three independent experiments ± standard deviation. Different accessibility values are colour coded as indicated. HMX profiles shown in the bottom panel correspond to difference in NMIA reactivity between the monomeric and the dimeric conformations (M-D) for the I+CU transcript. HMX scores indicated at precise positions were calculated from the reactivity profiles of the monomeric and dimeric isoform, as previously described^54^. (**b**) Sequence and secondary structure of I+CU, summarizing the HMX results. Filled circles: increase (>0.25 units) in NMIA relative reactivity for the monomer (M) compared to the dimer (D), with HMX score >0.55; open circles: increase (>0.25 units) in NMIA relative reactivity for the dimer (D) compared to the monomer (M), with HMX score >0.55.

**Figure 6:**
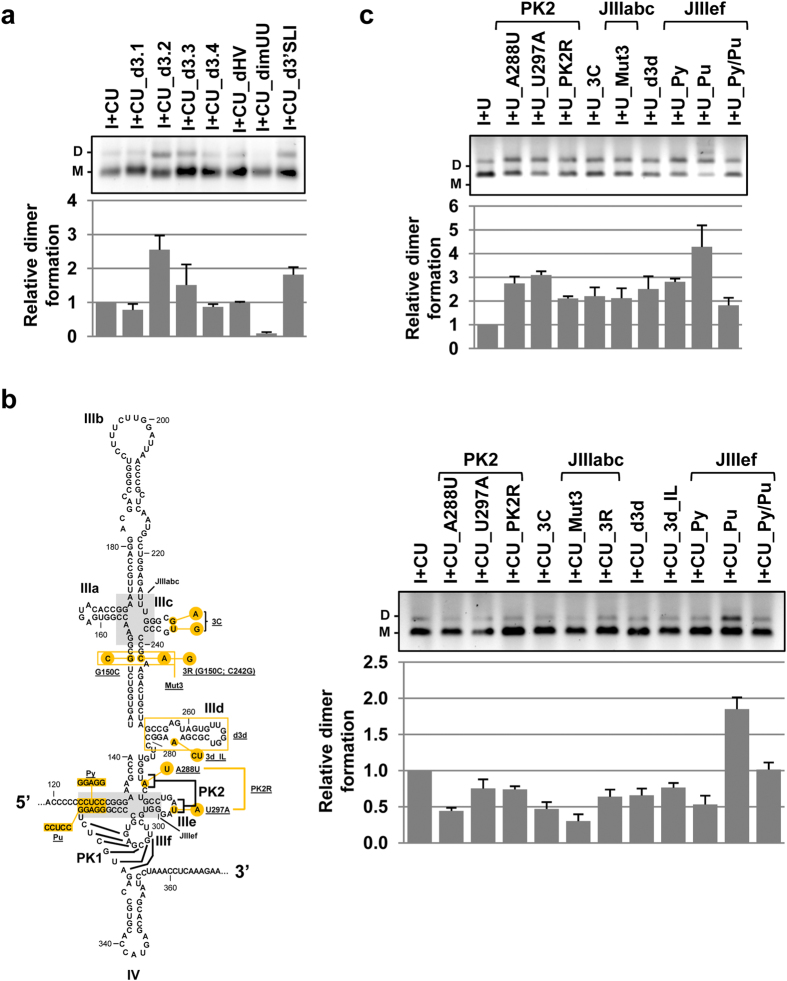
The IRES and the CRE control HCV dimerization efficiency. Data obtained from HMX assays were used to design a series of variants bearing different deletions or single-point mutations within the CRE and IRES. (**a**) Dimerization efficiency was monitored for deletion mutants of different RNA domains located throughout the CRE and the 3′UTR for I+CU. A defective dimerization mutant, the so-called_dimUU molecule^32^ containing the point A9539U mutation in the DLS motif, was employed as a negative control. Dimeric and monomeric conformers were resolved by native agarose gel electrophoresis. The relative dimerization yield for each molecule tested was quantified and normalized to that obtained for the non-mutated control transcript. Data are the mean of at least three independent experiments ± standard deviation. (**b,c**) Single-point mutations were introduced in the IRES region as indicated, for both I+CU (**b**) and I+U (**c**). The effect on dimer formation was evaluated and quantified as noted above. Values represent the mean of three independent assays ± standard deviation.

HMX analysis of the I+CU indicate that the required nucleotides in the 3′X-tail were dispersed throughout the entire region (Fig. 5). Interestingly, a number of residues in the 3′SLI domain (G9576, U9580, A9582 and A9603) were identified as critical partners, with an average HMX score of 0.63 ± 0.08 (Fig. 5). This result is consistent with the observation that the 3′SLI domain negatively affects kissing complex formation at the DLS motif (Fig. 6a and Supplementary Fig. S1). In addition, nucleotides G9295, U9296 and G9297, belonging to the internal loop of the 5BSL3.2, showed a considerable reduction in their NMIA sensitivity in the dimeric form with respect to the monomer conformation. The dimeric complex also showed differences in the SHAPE reactivity pattern for the internal loop of the IIId subdomain of the IRES region, plus other slight variations in the apical loop (Fig. 5b), with respect to the monomeric form. This is in good agreement with the idea of a direct role for the IRES:CRE interaction in the acquisition of a dimerizable conformation^43^. An increase in the reactivity for the dimer conformers was observed in the nucleotides surrounding the stop codon, pointing to a functional link between dimerization and translation. This hypothesis is further supported by the discovery of hot HMX points for dimerization in the IRES element (Fig. 6b), many of them located in essential regions for eIF3 and 40S ribosomal subunit recruitment, i.e., the IIIabc four-way junction^18^ and PK1-2^15^,^55^,^56^,^57^ respectively. These observations highlight the importance of long-distance RNA-RNA interactions in the three-dimensional RNA architecture and confirm that HCV genomic dimerization is controlled by RNA domains distinct from the 3′X-tail. More importantly, this finding underscores a functional link between viral protein synthesis and dimerization.

### Conserved structural elements in the CRE and the IRES regions control HCV dimerization efficiency

A series of mutants derived from the I+CU molecule was constructed based on the HMX results and thus determine which positions were biologically relevant in dimer formation.

In a first attempt, deletions of the different stem-loop elements located in the CRE and the 3′X-tail, with the exception of the essential 3′SLII and 3′SLIII domains, were designed (Methods). This design was due to the disperse pattern of the identified nucleotides, which hindered the construction of punctual mutants. Transcripts held the dimerization yield to that shown by the I+CU molecule (Fig. 6a), with the exception of deletions affecting domains 5BSL3.2 and 3′SLI. These data are in good agreement with previous HMX results pointing to domain 5BSL3.2 as a core partner in the dimer formation event, either by its interaction with the IRES or via a direct effect on 3′X-tail.

The role of specific positions of the IRES region in dimer complex formation was examined by generating nucleotide substitutions or deletions based on the data acquired using the HMX and/or SHAPE-dif strategies. For that aim, those regions bearing sets of nucleotides with HMX scores > 0.65 were identified as relevant structural elements and subjected to punctual mutations according to both sequence and conformational criteria, as reported^13^,^15^,^18^,^58^. A series of I+CU variants (Fig. 6b, left panel) was generated as described to perform the dimerization assays. Quantification of the dimerization efficiency revealed variation in the influence of the different mutations (Fig. 6b, right panel), though the yield of dimer formation was always affected. This result confirms the potential of structural analysis for identifying essential partners in the biological functions of RNA molecules. Several conclusions may be drawn. First, the folding of the IIIef junction must be preserved. Mutations disturbing the three-dimensional organization of this element^57^,^59^ result in defective (Py molecule) or improved (Pu molecule) dimer formation. Restoring the interactions (I+CU_Py/Pu variant) recovers non-mutated I+CU dimerization levels (Fig. 6b). Second, both the sequence and structure of the PK2 element are needed for dimerization (Fig. 6b). Third, the four-way junction IIIabc is critical for the generation of dimeric isoforms (see constructs I+CU_Mut3 and I+CU_3R; Fig. 6b). To our knowledge, this is the first report describing structural and sequence elements of the IRES region with a role in HCV genomic dimerization.

To determine whether the effects of IRES mutations are dependent on the presence of the CRE or directly affect the 3′X-tail, a new series of variants derived from I+U was designed. Surprisingly, all the mutations, by themselves, enhanced dimer formation from 2 to 4 fold (Fig. 6c) with respect to the I+U molecule. It might occur that such punctual mutations could promote conformational rearrangements in the IRES and thus avoid potential long-distant structural constraints for the acquisition of a dimerizable isoform, Further, this result clearly contrasts with that obtained for the IRES mutants derived from the I+CU mutant series (compare Fig. 6b and c), suggesting that the regulation of the HCV dimerization process is sustained by both the CRE and the IRES elements.

## Discussion

There is growing interest in understanding the role of the HCV genomic RNA dimerization^29^,^31^,^32^,^33^,^34^,^40^,^43^. One attractive hypothesis states that HCV dimer formation acts as a functional signal directing the switch between viral translation and replication. To explore this idea, the potential interactions of the DLS minimal dimerization motif with other regions of the viral genome need to be known. This paper reports that specific nucleotide positions located throughout the HCV IRES and CRE regions are involved in the regulation of dimer formation.

Electrophoretic mobility shift assays showed, for first time, the contribution of the distant IRES and CRE regions to dimer formation efficiency (Fig. 2). While the CRE improved dimerization yield compared to that shown by the 3′X-tail alone, the IRES region exerted an inhibitory effect that was quite patent even in the presence of the CRE enhancer. These observations suggest undiscovered IRES:3′X contacts, and place the IRES at the heart of the HCV dimerization regulatory pathway. The fact that essential functional regions such as the CRE and the IRES exert strong control over dimer formation suggests that this phenomenon is important for virus propagation. In addition, the IRES and CRE have roles in HCV translation^23^ and replication^35^,^36^, supporting the hypothesis that genomic dimerization is involved in the switch between those processes.

The present results go beyond previous proposals that the DLS motif is the only one required for dimer formation^29^,^31^. Though the DLS is indeed essential, the efficiency of dimerization is governed by distant genomic RNA elements (Fig. 2). Kissing complex formation is, *a priori*, only determined by the interaction of two DLS motifs; however, the acquisition of a dimerizable conformation, the stability of the dimer, and/or the yield of the reaction, are actually regulated by distant conserved domains in the IRES and CRE regions. Figure 2b shows that the addition of core chaperone protein allows a dimerization-competent structure to be achieved, since it improves 3′X construct dimer stability^29^. This effect occurs via the core protein binding to the single-stranded regions during the formation of the kissing complex, thereby avoiding intramolecular annealing and favouring extension towards a thermodynamically stable duplex. Significant dimerization yield improvement was not detected for CU, I+CU or I+U in the presence of the core protein (Fig. 2b), suggesting that additional, long-range RNA-RNA contacts were present in the dimers under study. Furthermore, significant variations in the stability of the dimeric forms were seen depending on the presence of IRES and/or CRE (Fig. 2c). Thus, the IRES and the CRE might be considered chaperone-like partners that aid and preserve the acquisition of the three-dimensional structure at the 3′ end of the viral genome.

Several hypotheses can be proposed about the mechanism by which the IRES and the CRE accomplish dimer formation control. It seems likely that the long-distance RNA-RNA interaction between the subdomain IIId and the 5BSL3.2 element^41^ is directly involved in the regulation of dimerization. The direct interaction between the IRES and the CRE was confirmed by SHAPE-dif analysis and supported by the observation that a number of nucleotides in the IRES account for differences in the reactivity profiles of I+CU compared to I+U (Figs 3b and 4). Importantly, some of the most prominent variations occur in subdomain IIId, the 5′ partner in the long-range contact IRES:CRE^41^. HMX identification of the nucleotides involved in dimer formation showed that, in the dimeric isoform, the bulge of the 5BSL3.2, and several residues of the subdomain IIId, show reactivities different to those seen in the monomer (Fig. 5). The HMX data also support the interaction 5BSL3.2–3′SLII in the I+CU RNA molecule (Fig. 5b), but not in the dimerizable competent construct CU (Supplementary Fig. 2), thus confirming that this connection must be repressed to induce the exposure of the DLS motif in the dimerizable isoform^29^,^32^. This observation apparently differs from ideas expressed in previous hypotheses^43^ in which it was proposed that the interaction IRES:CRE permits the acquisition of a dimerizable isoform. It should be remembered that in the analyses associated with these earlier ideas, suboptimal dimerization conditions (low RNA concentration) were used, with most of the molecules folded in monomeric form. It seems likely that increasing the RNA concentration influences the folding kinetics of the RNA transcripts^60^.

SHAPE-dif and HMX structural studies revealed the existence of a set of potential structural intermediates for the 3′X-tail (both in the monomeric and dimeric conformers; Figs 3b, 5 and Supplementary Fig. 2). Such different conformations can be acquired by the presence of specific residues within the IRES and the CRE, which would differentially interact with the 3′X-tail and/or other related regions. To our knowledge, no direct, stable RNA-RNA interaction between the 5′ end and the 3′X-tail of the HCV genome has ever been identified, though a reciprocal, fine-tuning conformational effect mediated by the IRES and the 3′UTR has been reported, which might face partial competition from other genomic regions such as the CRE^42^,^43^ (Figs 3b and 4). It is feasible that the generation of certain structural metastable and irreversibly misfolded intermediates would render inefficient dimerizable I+U or I+CU molecules, suggesting the IRES to be an agent that interferes with dimer formation. This idea is sustained by the HMX results and ensuing comparison of the data obtained for CU and I+CU (Fig. 5 and Supplementary Fig. 2). These results even show differences between the monomeric forms of both constructs at the 3′X-tail (compare monomer profiles in Fig. 5a and Supplementary Fig. 2; see also Supplementary Fig. S3A), suggesting direct contact is made between both termini of the HCV genome. In the dimer conformation, variations are also clear (Fig. 5a; Supplementary Figs 2 and S3B). This suggests that the dimer itself adopts different folding conformations depending on the structural and molecular environment. This correlates with the changes in dimer stability observed for the different constructs discussed above. In addition, the “misfolding hypothesis” is also supported by the results shown in Fig. 6c. These assays show that the introduction of mutations disturbing the architecture of the IRES region promote an increase in dimer formation efficiency, most likely by altering the structural dynamics of the 3′UTR. This tight relationship can be due the existence of long-range contacts between both ends of the viral genome, as previously proposed^42^,^43^. Further in-depth analysis is required to determine the biochemical and functional features involved.

This structural data were confirmed by dimerization assays of a series of RNA molecules bearing mutations for the IRES and CRE (Fig. 6). The results reveal the potential of the structural strategy for identifying those nucleotides that take part in HCV dimer formation. Interestingly, the identification of residues within the IRES controlling dimerization efficiency (Fig. 6b) provides definitive support for the idea of IRES-mediated chaperone-like activity in 3′X-tail conformation. This could be the result of local conformational rearrangements in the 3′X-tail dependent on the IRES. It has been shown that HCV genome is three-dimensionally organized in highly stable, constrained regions, with shape and plasticity defined by distant elements in the sequence, the so-called GORS (genome-scale ordered RNA structures)^61^,^62^,^63^. In this context, it seems feasible that the IRES could operate as destabilizing agent of the viral RNA geometry, an effect that can be extracted from data shown in Fig. 2c. Variations in global genome architecture, defined by the selective availability of different RNA functional elements, would control the dimerization event.

The regions identified in this work as dimerization regulatory elements play essential roles in the viral cycle. The essential IIIabc four-way junction is critical for eIF3 recognition during the initiation of IRES-dependent translation^18^. This work provides evidence to show that this junction is also important in regulating genomic dimer formation (Fig. 6b). Similar effects can be observed with respect to the changes affecting the PK2 element, particularly at the essential residue A288 (Fig. 6b). The PK2 motif helps to position the start translation codon at the P site in the 40S ribosomal subunit^15^. The structural role of the JIIIef motif in preserving HCV dimerization efficiency is also worth mentioning (Fig. 6b). This is a conserved element within the HCV genome that aids in the nucleation of the PK1 and aligns domains II and III on either side of a four-way junction, thus placing domain IV in the correct orientation in the 40S ribosomal subunit^16^. Another important element for regulating IRES function is domain 5BSL3.2 in the CRE region^23^. This interferes with HCV protein synthesis via its interaction with the essential apical loop IIId in the IRES^23^,^41^. The present results also highlight its involvement in the regulation of dimer formation (Fig. 6a). The essential role of 5BSL3.2 during viral replication^35^,^36^,^37^ and its interaction with 3′SLII, which contains the DLS motif, places dimerization at the heart of the pathway controlling the switch from translation to HCV RNA synthesis.

In addition to all the well-known functions of the IRES and CRE, a further role as RNA folding assistants can now be proposed; both elements appear to participate in guiding the dynamic shape of the HCV RNA genome. It may be that dimer formation modulates translation efficiency by swapping between different structural intermediates in the 80S-HCV RNA complex^64^. This might also promote the transition towards replication, in which dimerization is essential^34^. It should be also noted that the three partners involved in the dimerization process, i.e., the IRES, the CRE and the 3′UTR, are involved in the recruitment of viral and cellular factors^27^,^65^,^66^,^67^,^68^, which are related to viral protein and RNA synthesis processes. This points again to an interconnection between the dimer formation event and the transitions between viral translation and replication. It seems likely that selective binding of different proteins would determine the exposition of certain RNA motifs within the viral genome, which could unleash the establishment of inter- and intramolecular RNA-RNA interactions involved in the regulation of different steps during the viral cycle. Further work is needed to determine the precise molecular mechanism underlying this finely tuned, complex pathway.

Taken together, the present data provide key information on dimer formation requirements. They also support a novel role for the IRES and the CRE as chaperone-like partners, which influence, either directly or indirectly, the folding of distant functional elements in the HCV genome by establishing a complex network of contacts that control important stages of the HCV cycle.

## Methods

### DNA templates and RNA synthesis

DNA constructs encoding I+CU, I+U and CU (Fig. 1b) were obtained as previously described^43^. DNA molecules containing the 3′X-tail region (T7p3′X) (Fig. 1b) were obtained via the amplification of the plasmid pU3′HCV-9181, as previously reported^41^.

A series of plasmids containing different point mutations or deletions in the IRES, CRE or 3′UTR, were generated from the construct pGLI+CU^43^ by site-directed mutagenesis using the Phusion Site-Directed Mutagenesis Kit (Finnzymes). The primers used were (see Supplementary Table 1 for oligonucleotide sequence information): HCV-120mut125 and asHCV-119 for pGLI+CU_PolyPy; 3cBiloopAG and asHCV-228 for pGLI+CU_3c; HCV_Inser275CT and asHCV-252 for pGLI+CU_dIL3d; HCV-A288U and asHCV-276 for pGLI+CU_A288U; U297A and asHCV-276 for pGLI+CU_U297A; A288U/U297A and asHCV-276 for pGLI+CU_PK2R; HCV-311mut319 and asHCV-310 for pGLI+CU_PolyPu; HCV-9260 and asHCV-9215 for pGLI+CU_d3.1^23^; HCV-9311 and asHCV-9262 for pGLI+CU_d3.2^23^; HCV-9352 and asHCV-9320 for pGL-ICU_d3.3^23^; HCV-HCV-9358 and asHCV-9383 for pGLI+CU_d3.4; HCV-9507 and asHCV-9384muta for pGLI+CU_dHV; and dim_MutUU and HCV-9553 for pGLI+CU_dimUU.

The construct pGLI+CU_Py/Pu was generated by site-directed mutagenesis as described above using the oligonucleotides HCV-311mut319 and asHCV-310 plus the DNA template pGLI+CU_PolyPy.

Plasmid pGLI+CU_mut3 was obtained by two consecutive site-directed mutagenesis events. In the first step, a mutation was included at position 150 using the primers G150C and asHCV-140 with the DNA pGLI+CU. The resulting construct, pGLI+CU_G150C, was then subjected to a second round of site-directed mutagenesis by amplification with the oligonucleotides C242A and asHCV-228 to generate the plasmid pGLI+CU_mut3. The compensatory mutant, pGLI+CU_3R, was generated by amplification with the primers C242G and asHCV-228.

For the mutant series derived from the construct I+U, the same sets of oligonucleotides used for the I+CU-related series were employed in site-directed mutagenesis (see Supplementary Table 1).

DNA templates T7pI+CU, T7pI+CU_PolyPy, T7pI+CU_3c, T7pI+CU_dIL3d, T7pI+CU_A288U, T7pI+CU_U297A, T7pI+CU_PK2R, T7pI+CU_PolyPu, T7pI+CU_d3.1, T7pI+CU_d3.2, T7pI+CU_d3.3, T7pI+CU_d3.4, T7pI+CU_dHV and T7pI+CU_dimUU were obtained by amplification from their respective plasmids using the primers 5′T7pHCV^69^ and 3′HCV^41^ to achieve the precise 3′ termini. The construct T7pI+CU_d3′SLI was generated by PCR amplification using the DNA plasmid template pGLI+CU and the primers 5′T7pHCV and asHCV-9585.

Molecules encoding the templates for the transcripts CU, CU_d3.1, CU_d3.2, CU_d3.3, CU_d3.4, CU_dHV and CU_dimUU were derived from their respective DNA constructs by amplification with the oligonucleotides 5′T7pHCV-9181 and 3′HCV^41^. The resulting RNA molecules bore the correct 3′ ends. Template T7pCU-d3′SLI was constructed from plasmid pGLI+CU by PCR using the primers 5′T7pHCV-9181 and asHCV-9585.

For differential SHAPE assays (SHAPE-dif) and 2′-hydroxyl molecular interference (HMX) analyses, DNA templates coding for the transcripts I+CU, CU and I+U were obtained by attaching a 45 nt cassette to their 3′ termini^43^,^51^. This cassette neither interferes with the structural dynamics of the HCV 3′UTR nor with dimerization efficiency (data not shown), but provides an annealing site for efficient primer extension. Briefly, molecules T7pI+CU, T7pCU and T7pI+U encompassing this cassette were obtained from the plasmid pGLI+CU (for T7pI+CU and T7pCU) or pGLI+U^43^ (for T7pI+U) as previously reported^43^: primers 5′T7pHCV and 3′HCV_cassette generated the DNA template T7pI+CU_cassette or T7pI+U_cassette, while amplification with oligonucleotides T7pHCV-9181^41^ and 3′HCV-cassette yielded the construct T7pCU_cassette.

RNA synthesis was accomplished using the TranscriptAid T7 High Yield Transcription Kit (ThermoFisher Scientific), following the manufacturer’s instructions. The resulting RNA products were purified as previously described^23^. The quality of the transcripts and their concentration was monitored as reported^23^.

### Core peptide

The chimeric peptide 2BD, with the sequence PRRGPRLGVRATRKTSERSQPRGRRQPIPKVRHQTGRRGSRPNWGPNDPRRRSRNLGK, was chemically synthesized and purified by the American Peptide Company.

### RNA dimerization assays

Increasing amounts (0.1–5 μM) of the RNA molecules 3′X, CU, I+U and I+CU were incubated in SHAPE buffer (20 mM HEPES pH 8.0, 50 mM NaCl, 1 mM MgCl_2_)^42^ at 37 °C for 15 min. The resulting complexes were then resolved by native gel electrophoresis (10% polyacrylamide gels for the 3′X molecule and 1–1.5% agarose gels for CU, I+U and I+CU molecules) in TBM buffer (45 mM Tris-HCl pH 8.3, 43 mM boric acid, 0.1 mM MgCl_2_)^41^ at 4 °C. Electrophoresis was performed over 3–5 h at 12 mA (for polyacrylamide gels) or 25 mA (for agarose gels). The reaction products were visualized by staining with RedSafe^TM^ (Ecogen), and quantified using Image Lab^TM^ software (Bio-Rad).

The contribution of the 2BD peptide to dimer formation was analyzed by adding it to binding reactions (at a protein:nucleotide molar ratio of 1:50), followed by incubation at 37 °C for 15 min. The protein:nucleotide saturation ratio was established at 1:50. These samples were subsequently treated with proteinase K (300 ng/μl) in the presence of 0.5% SDS for 30 min at 37 °C. RNA molecules were then purified by phenol extraction and resolved in 4–10% native polyacrylamide gels as described above.

Melting curves were produced by incubating 1 μM of the transcripts 3′X, CU, I+U or I+CU in SHAPE buffer. The melting temperatures ranged from 30 °C to 80 °C. Reactions proceeded for 15 min; the RNA molecules were then electrophoresed as described above.

The RNA dimerization capacity of the IRES and CRE mutants was essentially performed as described above, using 1 μM of each RNA construct.

### Differential SHAPE assays

SHAPE-dif assays were essentially performed as previously described^50^. Briefly, 25 pmol of the RNA molecules under study were folded in SHAPE buffer by incubation at 37 °C for 5 min (optimal for dimer formation). After folding, the RNA constructs were probed with 15 mM of NMIA (N-methyl isatoic anhydride) or 16 mM of 1M6 (1-methyl-6-nitroisatoic anhydride). Reactions proceeded for 20 min and 5 min respectively at 37 °C. Control samples containing no reagent were prepared in parallel with an equivalent volume of DMSO. Modification was stopped with the addition of cold 300 mM sodium acetate at pH 5.2. The RNA was then ethanol-precipitated, washed three times with 80% ethanol, and quantified by UV spectrophotometry (A_260_). The formation of 2′-*O*-adducts with both NMIA and 1M6 was mapped by primer extension using fluorescently-labelled oligonucleotides. Briefly, primer extension reactions were performed with 2 pmol of modified RNA and 20 U of SuperScript III RT (Invitrogen, Life Technologies) using the following fluorescently-labelled primers: oligonucleotide Std^43^ (which hybridizes within the cassette motif at the 3′ end of the RNA constructs and allows for reading of the 3′ end of the HCV genome), and asHCV-372 and asHCV-539^42^ (employed for mapping the IRES region). NED-labelled oligonucleotides were used to detect both untreated and positive reactions; FAM and VIC fluorophores were chosen for detecting sequencing ladders. A fraction of the resulting cDNA samples was purified as described^42^. Electrophoretograms were analyzed using QuShape software^70^. Normalized reactivity values at each nucleotide position were calculated by dividing each value by the average intensity of the 10% most reactive residues, after excluding outliers^42^. 1M6 reactivities were then scaled, using the implemented differential SHAPE algorithm^71^, to those obtained for NMIA treatments by minimizing the reactivity difference over a 25 nt sliding window. The scaled reactivities were then subtracted from the NMIA values to create the differential SHAPE profiles.

### 2′-hydroxyl molecular interference (HMX) assays

HMX analysis was performed as previously described^54^. 100 pmol of each RNA construct was subjected to NMIA modification under denaturing conditions. For this, 30 pmol of each RNA RNA were first heated at 95 °C for 2 min in 18 μl of 100 mM HEPES pH 8.0, and then cooled at 4 °C for 15 min. RNA modification was initiated by adding NMIA 15 mM. Reactions proceeded for 3 min at 95 °C before cooling on ice. This modification process was repeated twice. The evaporated water was replaced to reach the final target volume. The modified molecules were precipitated with ethanol and washed twice with 80% ethanol. The amount of RNA was monitored by UV spectrophotometry (A_260_). Dimer formation was accomplished as described above by incubating 1 μM of each RNA variant in SHAPE buffer for 15 min at 37 °C. Complexes were resolved by polyacrylamide non-denaturing gel electrophoresis (4–6%) in TBM buffer and visualized by UV shadowing. The specific reaction products corresponding to the monomeric or dimeric isoforms were excised from the gel and passively eluted overnight at 4 °C. NMIA modifications were detected by primer extension and the cDNA products analyzed by capillary electrophoresis, as described above. HMX scores were calculated as previously described^54^.

## Additional Information

**How to cite this article**: Romero-López, C. *et al*. The chaperone-like activity of the hepatitis C virus IRES and CRE elements regulates genome dimerization. *Sci. Rep.* **7**, 43415; doi: 10.1038/srep43415 (2017).

**Publisher's note:** Springer Nature remains neutral with regard to jurisdictional claims in published maps and institutional affiliations.

## Supplementary Material

Supplementary Data
