# Supplementary material for: The chaperone-like activity of the hepatitis C virus IRES and CRE elements regulates genome dimerization
Source: Sci Rep. 2017 Feb 24;7:43415. doi: 10.1038/srep43415 (PMC5324077; doi:10.1038/srep43415)

**SUPPLEMENTARY DATA**

# The chaperone-like activity of the hepatitis C virus IRES and CRE elements regulates genome dimerization

Cristina Romero-López, Alicia Barroso-delJesus and Alfredo Berzal-Herranz

**Supplementary Table 1.** Oligonucleotides used to construct DNA plasmids described in this study.

| Construct | Oligonucleotides | Sequence (5’-3’) |
|---|---|---|
| **pGLI+CU_A288U** | A288U | GGCCTTGTGGTTCTGCCTGATAGGGT |
| | asHCV-276 | TTTCGCGACCCAACACTACTCGGCTA |
| **pGLI+CU_U297A** | U297A | GGCCTTGTGGTACTGCCTGAAAGGGT |
| | asHCV-276 | TTTCGCGACCCAACACTACTCGGCTA |
| **pGLI+CU_PK2R** | A288U/U297A | GGCCTTGTGGTTCTGCCTGAAAGGGT |
| | asHCV-276 | TTTCGCGACCCAACACTACTCGGCTA |
| **pGLI+CU_G150C** | G150C | TAGTGGTCTCCGGAACCGGTGAGTA |
| | asHCV-140 | TGGCTCTCCCGGGAGGGGGGGTCCTGGA |
| **pGLI+CU_Mut3** | C242A | GGGCGUGCCCCCGAAGACTGCTA |
| | asHCV-228 | AAATCTCCAGGCATTGAGCGGGTT |
| **pGLI+CU_3R** | C242G | GGGCGTGCCCCCGGAAGACTGCTA |
| | asHCV-228 | AAATCTCCAGGCATTGAGCGGGTT |
| **pGLI+CU_3c** | 3c_BiloopAG | GGGCAGGCCCCCGCAAGACTGCTA |
| | asHCV-228 | AAATCTCCAGGCATTGAGCGGGTT |
| **pGLI+CU_d3d** | asHCV-252 | TAGCAGTCTTGCGGGGGCACGCCCAA |
| | HCV-284 | GTGGTACTGCCTGATAGGGTGCTTGCGAGT |
| **pGLI+CU_dIL3d** | HCVInser_275CT | GCCGAGTAGTGTTGGGTCGCGACTAGGCCTT |
| | asHCV-252 | TAGCAGTCTTGCGGGGGCACGCCCAA |
| **pGLI+CU_Pu** | HCV-311mut319 | GTGCCCCGCCTCCTCTCGTAGACCGT |
| | asHCV-310 | TCGCAAGCACCCTATCAGGCAGTA |
| **pGLI+CU_Py** | HCV-120mut125 | CCCCCGGAGGCGGGAAAGCCATAGT |
| | asHCV-119 | TCCTGGAGGCTGCACGACACTCAT |
| **pGLI+CU_Py/Pu** | HCV-311mut319 | GTGCCCCGCCTCCTCTCGTAGACCGT |
| | asHCV-310 | TCGCAAGCACCCTATCAGGCAGTA |
| **pGLI+CU_d3.1** | HCV-9260 | TTACAGCGGGGGAGACATATATCACAG |
| | asHCV-9215 | GATTGGAGTGAGTTTGAGCTTGGTCCT |
| **pGLI+CU_d3.2** | HCV-9311 | GTTCATGTGGTGCCTACTCCTACTTTCTGTA |
| | asHCV-9262 | TAACCAGCAACGAACCAGCTGGATAAATCCAA |
| **pGLI+CU_d3.3** | HCV-9352 | TCTATCTACTCCCCAACCGATGAACGG |
| | asHCV-9320 | CCACATGAACCAGCGGGGTCGGGCA |
| **pGLI+CU_d3.4** | HCV-9384 | TAAACACTCCAGGCCAATAGGCCA |
| | asHCV-9358 | GCTCCCCGTTCATCGGTT |
| **pGLI+CU_dHV** | HCV-9507 | TATGGTGGCTCCATCTTAGCCCTA |
| | asHCV-9384muta | AGCTCCCCGTTCATCGGTTGGGGAGT |
| **pGLI+CU_dimUU** | HCV-9553 | CGTGAGCCGCATGACTGCAGAGA |
| | Dim_Mut_UU | GACCTTTCACAGCAAGCCGTGATTA |
| **pGLI+CU_d3’SL1** | 5’T7pHCV | TAATACGACTCACTATAGGGATGGAAGCCAAAAACA |
| | asHCV-9560 | GGCTCACGGACCTTTCACAGCTA |

**Supplementary Table 2.** Oligonucleotides used in this study.

| Oligonucleotide | Sequence (5'-3') |
|---|---|
| **5'T7pHCV** | TAATACGACTCACTATAGCCAGCCCCCTGATGG |
| **5'T7pHCV-9181** | TAATACGACTCACTATAGGGCAGTAAGGACCAAGCTCAA |
| **3'HCV** | ACTTGATCTGCAGAGAGGCCA |
| **3'HCV_cassette** | GAACCGGACCGAAGCCCGATTTGGATCCGGCGAACCGGATCGAACATGATCTGCAGAGAGGCCAGTA |
| **asHCV-9585** | GTATCAGCACTCTCTGCAGT |
| **Primer Std** | GAACCGGACCGAAGCCCG |

Promoter sequence for the T7 RNA polymerase is underlined.

**Supplementary Figure S1.** The 3’SLI interferes with the efficient dimer formation. Dimerization efficiency was assayed at increasing concentrations of 3’X and X55, which contains only the 3’SLIII and 3’SLII domains required for HCV genomic dimerization (40). Dimeric products were resolved by native TBM polyacrylamide gel electrophoresis. A representative image of the electrophoretic mobility shift assays is shown. Relative dimer formation for each transcript was quantified. M, monomer; D, dimer.

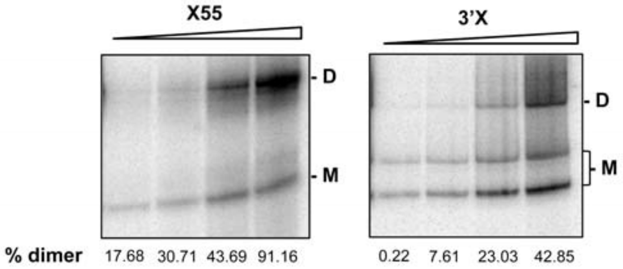

**Supplementary Figure S2.** Identification of nucleotides influencing dimer formation in the CRE region of the HCV genome. Nucleotides required for dimer formation were identified by 2'-hydroxyl molecular interference (HMX). A) Molecule CU was modified with NMIA under denaturing conditions. The monomeric and dimeric populations were then partitioned by native polyacrylamide gel electrophoresis. Modified positions were detected as stops in a reverse transcription reaction. Histograms show the NMIA reactivity profile for each of the isolated pools, M (monomer) and D (dimer). Data are the mean of three independent experiments ± standard deviation. Different accessibility values are colour coded as indicated. HMX profiles shown in the bottom panel correspond to difference in NMIA reactivity between the monomeric and the dimeric conformations (M-D) for the CU transcript. HMX scores indicated at precise positions were calculated from the reactivity profiles of the monomeric and dimeric isoform, as previously described {Homan, 2014 #2794}. B) Sequence and secondary structure of CU, summarizing the HMX results. Filled circles: increase (>0.25 units) in NMIA relative reactivity for the monomer (M) compared to the dimer (D), with HMX score >0.55; open circles: increase (>0.25 units) in NMIA relative reactivity for the dimer (D) compared to the monomer (M), with HMX score >0.55.

A)

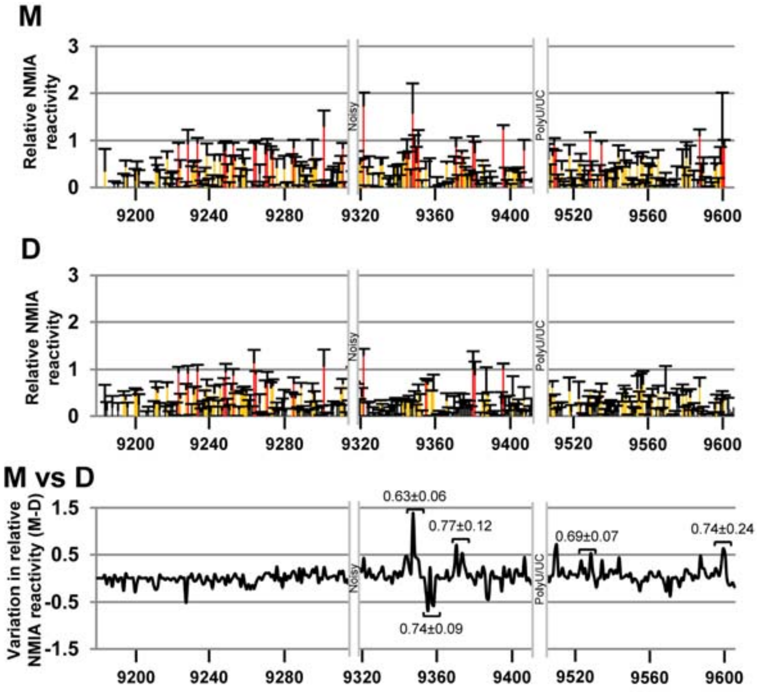


B)

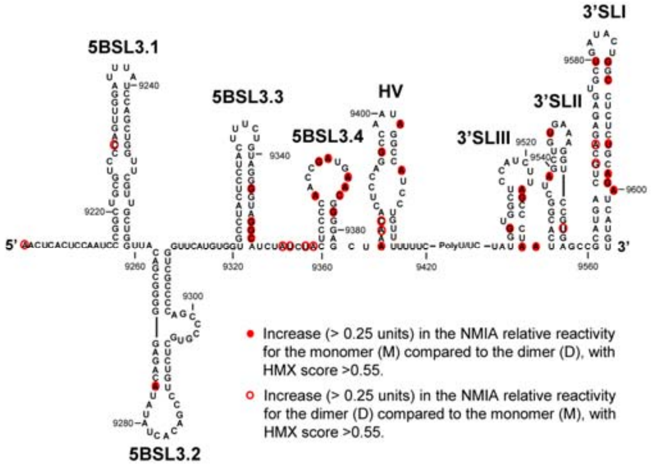

**Supplementary Figure S3.** Line graph showing the differential SHAPE reactivities for the transcripts CU and I+CU calculated by scaling 1M6 to NMIA relative values and then subtracting corrected 1M6 from NMIA reactivity data, under monomeric (A) or dimeric (B) conditions.

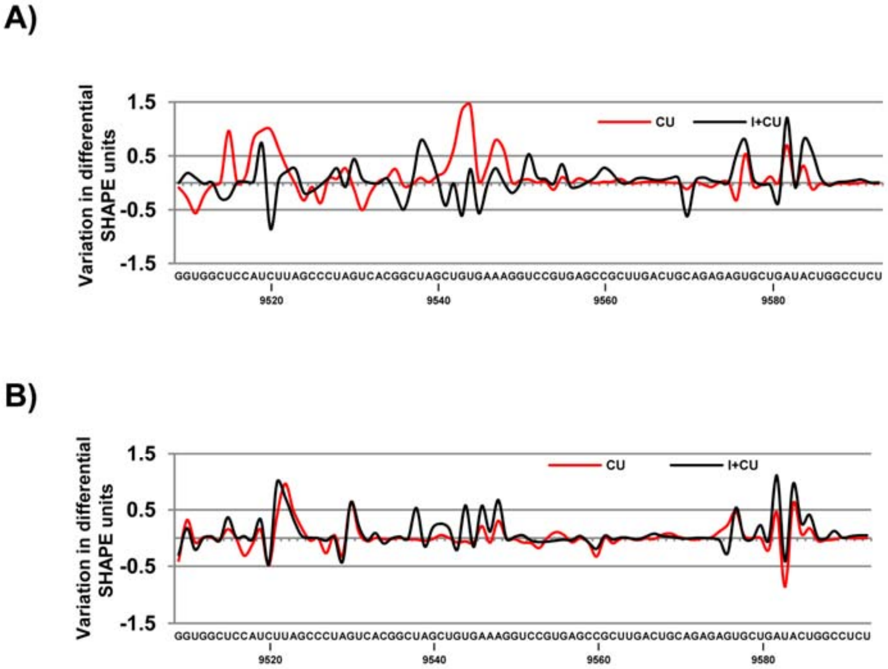

Supplement: Supplementary Data [file srep43415-s1.pdf]
